# Anxiety and depression among perinatal women during the long-term normal prevention of COVID-19 pandemic period in China: a cross-sectional study

**DOI:** 10.1186/s12888-023-04930-6

**Published:** 2023-06-21

**Authors:** Weiping Chen, Wei Peng, Yan Zhang, Huansheng Zhou, Meng Zhang

**Affiliations:** 1 Department of Gynecology, the Affiliated Hospital of Qingdao University, Qingdao, China; 2 Department of Obstetrics, the Affiliated Hospital of Qingdao University, Qingdao, China

**Keywords:** Anxiety, Depression, Perinatal, Pregnant women, COVID-19, Long-term normal pandemic prevention

## Abstract

**Background:**

COVID-19 has increased the probability of occurrence of maternal anxiety and depression in pregnant women. However, there is limited research on anxiety and depression among pregnant women during the long-term normal prevention of COVID-19 pandemic period. This study aimed to examine the anxiety and depression and influencing factors among perinatal women during the long-term normal prevention of COVID-19 pandemic period in China.

**Methods:**

A cross-sectional survey was designed. A total of 1338 pregnant women were studied. The prenatal anxiety and depression were assessed by the Self-rating Anxiety Scale (SAS) and the Self-rating Depression Scale (SDS), respectively. Postnatal depression was assessed by the Edinburgh Postpartum Depression Scale (EPDS) in 10–14 days after delivery. The data analysis was processed by SPSS9.0. Descriptive analysis was expressed by mean and standard deviation. The counting data were expressed by percentage, χ2 test, multiple linear regression and binary logistic regression.

**Results:**

The incidence of prenatal anxiety (SAS score ≥ 50) was 27.95% (374 cases), prenatal depression (SDS score ≥ 0.5) was 34.01% (455 cases), and postpartum depression (EPDS score ≥ 0.5) was 25.04% (335 cases). Both the prenatal SAS score (r = 0.635, P < 0.001) and prenatal SDS score (r = 0.738, P < 0.001) were related to postpartum depression. Pregnant women who were younger than 35 years, in middle household income, lower education level, underweight before pregnancy, primiparous, and fear of being infected were at increased risk for developing anxiety and depression during the long-term normal prevention of COVID-19 pandemic.

**Conclusions:**

The incidences of postpartum depression among perinatal women during the long-term normal prevention of COVID-19 pandemic period were a little lower than those during the COVID-19 outbreak period, but still higher than those before the COVID-19.

## Introduction

Coronavirus disease of 2019 (COVID-19) was a highly contagious respiratory tract infection disease caused by Severe Acute Respiratory Syndrome Coronavirus type 2 (SARS-CoV-2) resulting in a global pandemic status [1, 2]. Shortly after been recognized, the outbreak of COVID-19 was declared a public health emergency of international concern by World Health Organization [3]. The COVID-19 pandemic had profound effects on health-care systems, societal structures, and the world economy. Due to the characteristics of strong infectivity, large number of infected persons, high proportion of early critical cases and high mortality rate, COVID-19 caused people severe physical and mental trauma, especially to perinatal pregnant women [4–6].

Pregnancy, as a vulnerable population, is a particularly vulnerable time when psychological distress can have negative consequences for both mother and baby. Since women tend to report higher symptoms of anxiety and depression during disease outbreaks than men, pregnant women during the COVID-19 pandemic may be especially affected.[7, 8]. Pregnant women in the perinatal period were often accompanied by a series of physiological and psychological changes. Pregnant women may be of a particular concern, as anxiety has been described as a common psychological problem during pregnancy [9]. Postpartum depression was considered to be one of the most common psychiatric complications of childbirth and was a major cause of maternal mortality worldwide. Prenatal anxiety and depression symptoms may also cause changes in physical activity, nutrition, and sleep, which in turn affect maternal mood and fetal development. Anxiety and depression during pregnancy increased the risk for miscarriage, preterm birth, lower birth weight, lower Apgar score, and fetal death [10]. Children of mothers who experienced high stress and depression during pregnancy are more likely to have cognitive and behavioral problems, and are at higher risk for later mental health problems [11, 12]. Thus, pregnant women, as a group, may be more likely to experience elevated depressive symptoms in response to the pandemic than are non-pregnant women, and among pregnant women, those who experience more severe stress and adversity may be at highest risk [13].

In recent years, maternal anxiety and depression during the perinatal period has been one of the key issues that obstetrics and gynecology and psychiatric medical staff focused on [14]. During the COVID-19 pandemic, nationwide lockdowns, disruption of health-care services, and fear of attending health-care facilities also affected the wellbeing of pregnant people and their babies. These increased the probability of occurrence of maternal anxiety and depression [15, 16]. There has been plenty of studies on the anxiety and depression among pregnant women during the outbreak of COVID-19 [17]. Previous research showed that anxiety and depression of pregnant women from US, Europe, Türkiye, and China would increase during the outbreak of COVID-19 [18–21]. Generally, anxiety and depression symptoms in pregnancy typically affect between 10 and 20% of pregnant individuals [7]. In China, pregnant women during COVID-19 outbreak had significantly higher rates of depressive symptoms (29.6%). The main risk factors for developing depressive and anxiety during the outbreak included underweight before pregnancy, primiparous, younger than 35 years, employed full time, in middle income category, and with appropriate living space were at increased risk symptoms [22]. In the early phase of the COVID-19 outbreak, pregnant individuals in Canada are experiencing substantially elevated anxiety and depression symptoms that are significantly related to COVID-19 specific worries about threats to their own lives, their baby’s health, not getting enough prenatal care, and social isolation [7]. Women who were pregnant in the San Francisco during the pandemic were nearly twice as likely to have possible depression than were matched women who were pregnant prior to the pandemic [23]. After the outbreak period of COVID-19, it entered a long-term normal prevention of COVID-19 pandemic period, which means an ordinary long-term normal state of pandemic prevention and control for a long period of time (several months or several years). In the long-term normal prevention period people can basically return to long-term normal life and work while taking protective measures. There were 3 different COVID-19 situations in China, i.e. the COVID-19 outbreak period (3 months from 2019 winter), the long-term normal prevention period (3 years from 2020 spring) and release the lock down and strict control (from 2022 December). The long-term normal prevention period lasted the longest time (nearly 3 years) and it greatly affected people’s life and mental health. Therefore, we chose this survey time period. However, there is limited research on the anxiety and depression among pregnant women during the long-term normal prevention of COVID-19 pandemic period. Therefore, it is urgent to explore the mental health and risk factors of depression among perinatal women during the long-term normal prevention of COVID-19 pandemic period in China.

In this study, a questionnaire survey was conducted to investigate the anxiety and depression among perinatal women, to clarify the main factors of the depression and provide effective treatment suggestions. It was helpful to provide strategies and reference for clinical intervention to reduce the depression of pregnant women during the long-term normal prevention of COVID-19 pandemic period. The highlight of this study included that this study examined the anxiety and depression of pregnant women and the corresponding risk factors during the long-term normal prevention of COVID-19 pandemic period. It not only studied the prenatal anxiety and depression, but also explored the postnatal depression. It filled up the gap that most research focused on the COVID-19 outbreak period and few reported the mental health of pregnant women during the relatively long-term normal prevention of COVID-19 pandemic period.

## Methods

### Participants

A total of 1338 pregnant women hospitalized to our hospital for delivery from January 1 to June 30, 2022 were selected as the research subjects. Inclusion criteria: ① be able to complete the questionnaire; ② prenatal examination indicators are normal; ③ no infection of COVID-19; ④ volunteer to participate in this survey. Exclusion criteria: ① severe pregnancy complications or comorbidity, repeated abortion, high-risk pregnancy; ② patients diagnosed with COVI-19; ③ with mental disorders and poor communication ability; ④ with depression before pregnancy, severe sleep apnea syndrome and sleeping pill dependence; ⑤ patients with past or present history of substance use.

### Questionnaire survey

This was a cross-sectional study through a self-administered questionnaire [24]. The questionnaire survey was used to investigate the related factors affecting maternal psychological status. The anonymous survey questionnaire was designed with three sections to collect data regarding: (1) background demographic, pregnancy status; (2) attitudes towards COVID-19; (3) anxiety and depression status. The content of the questionnaire was reviewed and pretested by professors in psychiatry and obstetrics.

### Demographic characteristics of participants

The demographic characteristics of the participants included in this questionnaire were developed by the research panel, which was composed by multidisciplinary specialists in obstetrics and psychiatry, including clinical doctors and psychologist. In addition, some authoritative research results and references were consulted [25, 26]. The demographic data of participants mainly included gestational age, place of residence, education level, annual family income, occupation, body mass index (BMI), parity (nullipara or multipara), gestational week, ways of conception, delivery mode, severe pain during delivery, comorbidity and complication (including gestational hypertension, gestational diabetes, intrahepatic cholestasis and cervical disease), sleep quality, family support, worrying side effects of prenatal chest CT examination on fetal development, fear of disrupted the scheduled pregnant inspection, worrying about difficulty in hospitalization, fear of being infected COVID-19, fear of losing the job positon after delivery, and knowledge of COVID-19 ,and so on.

### Prenatal anxiety and depression

The prenatal anxiety was assessed using Zung’ Self-rating Anxiety Scale (SAS)[27], which consisted of 20 items with 1, 2, 3 and 4 points for each item. The total raw score, i.e. the sum of each item, ranged among 20–80 points. The standard score was calculated using the total raw score multiplied by 1.25. A standard score < 50 points was not considered anxiety. A standard score ≥ 50 indicated anxiety status, i.e. standard scores of 50–59, 60–69 and ≥ 70 were considered mild, moderate and severe anxiety, respectively. The anxiety was more serious with the increasing standard score. The prenatal depression was evaluated by Zung’ Self-rating Depression Scale (SDS)[27]. The scale consisted of 20 items, each of which was assigned 1, 2, 3 and 4 points, and the total score of the items was 20–80 points. The depression severity degree was measured by the value of total score of each item divided by 80. It was considered as no depression when the total score was less than 0.5. It was considered depression only when the total score was 0.5 or above.

### Postpartum anxiety and depression

Edinburgh Postnatal Depression Scale (EPDS) was used to evaluate postpartum depression symptoms within 10–14 days after delivery [28]. The scale included 10 sub-items, with scores of 0, 1, 2 and 3 points for each item, with a total score of 0–30 points. The total score with more than 13 points was diagnosed as postpartum depression, and the higher the score, the more serious postpartum depression.

### Data analysis

The data statistical analysis was processed by IBM SPSS Statistics for Windows, version 19 (IBM Corp., Armonk, N.Y., USA). Descriptive analysis was expressed by mean and standard deviation. The counting data were expressed and analyzed by percentage, χ2 test, multiple linear regression and binary Logistic regression. Pearson linear correlation analysis was used to analyze the correlation between the two variables. P < 0.05 mean the difference was statistically significant [29].

All factors related to pregnant women’s background and their attitude towards COVID-19 were selected as independent variables. Multivariate linear regression analysis was used to determine the effect of these factors on the anxiety and depression status [30] .

### Ethical consideration

The study protocol was approved by the Ethics Committee of the Affiliated Hospital of Qingdao University, China. Relevant guidelines were followed to ensure that the study was voluntary and confidential. The informed consent was obtained with completing the questionnaire.

## Results

### Participants’ demographic characteristics

A total of 1338 parturient women were included in this study. The majority of the pregnant women were with age of younger than 35 (85.80%), university degree (64.05%), a middle annual family income (52.02%), BMI between 18.5 and 23.9 kg/m^2^ (64.87%), nullipara (70.85%), Spontaneous pregnancy (91.93%), and with good family support (68.98%). The detailed demographic and obstetrics characteristics of the participants was presented in Table 1.

**Table 1:** Demographic and obstetrics characteristics of participants (n = 1338)

Characteristics	Mean ± SD or N (%)
age (years) (Mean ± SD)	27–32 (31.50 ± 3.48)
<35	1148 (85.80)
≥ 35	190 (14.20)
place of residence	
urban areas	1046 (78.18)
rural areas	292 (21.82)
education level	
high school or below	188 (14.05)
university	857 (64.05)
post-graduate	293 (21.90)
annual family incomes (RMB)	
low (< 100,000)	409 (30.57)
middle (100,000-400,000)	696 (52.02)
high (>400, 000)	233 (17.41)
employment	
employed	990 (73.99)
unemployed	348 (26.01)
BMI median (range (kg/m^2^)	20.5 (19.2–22.8)
< 18.5	128 (9.57)
18.5–23.9	968 (72.35)
≥ 24	242 (18.09)
parity	
primiparous	948 (70.85)
multiparous	390 (29.15)
gestational week,	
≥ 38	1221 (91.26)
< 37	117 (8.74)
ways of conception	
natural	1230 (91.93)
assisted reproductive technology	108 (8.07)
delivery mode	
vaginal	616 (46.04)
cesarean	722 (53.96)
severe pain during delivery	663 (49.55)
comorbidity and complication	107(8.00)
poor family support	639 (47.76)
poor sleep quality	617 (46.11)
worrying side effects of prenatal CT	893 (66.74)
fear of disrupted the scheduled pregnant inspection	700 (52.32)
worrying about difficulty in hospitalization	438 (32.74)
fear of being infected COVID-19	1016 (75.93)
fear of losing the job positon after delivery	710 (53.06)
lack of knowledge of COVID-19	715 (53.43)

### Correlation between prenatal anxiety/depression and postpartum depression

During the long-term normal prevention of COVID-19 pandemic period, the incidence of prenatal anxiety was 27.95% (SAS ≥ 50, 374 cases), prenatal depression 34.01% (SDS ≥ 0.5, 455 cases), and postnatal depression 25.04% (EPDS ≥ 13, 335 cases). The correlation analysis between prenatal anxiety/depression and the occurrence of postpartum depression showed that the higher the prenatal SAS score was, the higher the risk of postpartum depression was. A significant positive correlation was found between prenatal anxiety and postpartum depression (r = 0.635, P < 0.001). In addition, the higher the prenatal SDS score, the higher the risk of depression. There also existed a significant positive correlation between prenatal depression and postnatal depression (r = 0.738, P < 0.001), which was shown in Table 2.

**Table 2:** Correlation between prenatal anxiety and depression and postpartum depression

item	Cases (%)	EPDS ≥ 13	EPDS < 13	postpartum depression (%)	r	P
SAS < 50	964 (72.05)	63	901	6.54	0.148	0.113
SAS ≥ 50	374 (27.95)	272	102	72.73	0.635	< 0.001
SDS < 0.5	883 (65.99)	36	847	4.08	0.251	0.016
SDS ≥ 0.5	455 (34.01)	299	156	65.71	0.738	< 0.001

### Related risk factors for prenatal anxiety and depression

We explored risk factors for prenatal anxiety and depression during the long-term normal prevention of COVID-19 pandemic. Compared with pregnant women without prenatal anxiety (SAS < 50), women were at increased risk for prenatal anxiety if they were those who with (1) an age younger than 35 years, (2) an education of below high school, (3) a low annual household income, (4) a BMI of less than 18.5 kg/m^2^, (5) a parity of nullipara, (6) an assisted reproductive technology, (7) pregnancy complications, (8) a delivery mode of cesarean, (9) severe pain, (10) poor sleep quality, (11) poor family support, (12) worrying about the adverse impact of prenatal chest CT examination, (13) fear of disrupted the scheduled pregnant inspection, (14) worrying about difficulty in hospitalization, (15) fear of being infected COVID-19, (16) fear of losing the job positon after delivery, and (17) shortage of knowledge of COVID-19. Those who had fever, cough, diarrhoea or symptoms of suspected infection were more likely to have anxiety than healthy women.

Compared with pregnant women without prenatal depression (SDS < 0.5), women at higher risk for depressive symptoms included those with age younger than 35 years, a BMI of less than 18.5 kg/m^2^, a below college education, a middle annual household income, a parity of nullipara, an assisted reproductive technology, pregnancy complications, fear of losing the job positon after delivery, fear of being infected COVID-19, disrupted the scheduled pregnant inspection, poor family support, poor sleep quality. The participants’ knowledge, attitudes, and practice regarding the COVID-19 pandemic seemed to easily impact the depressive symptoms during the long-term normal pandemic prevention of COVID-19. The analysis of related factors of antenatal anxiety and depression was shown in Table 3.

**Table 3:** Related factors of prenatal anxiety and depression [cases (%)]

	Prenatal anxiety						Prenatal depression		
	SAS < 50	SAS ≥ 50	χ2	P	SDS < 0.5	SDS ≥ 0.5		χ2	P
total	964 (72.05)	374 (27.95)	-	-	883( 65.99)	455 (34.01)		-	-
age: <35	810 (84.02)	338 (90.37)	18.02	< 0.001	717 (81.20)	431 (94.73)		30.16	< 0.001
education: high school or below	68 (7.05)	120 (32.09)	14.90	< 0.001	62 (7.02)	126 (27.69)		34.10	< 0.001
income: low	179 (18.57)	230 (61.50)	34.20	< 0.001	163 (18.46)	246 (54.07)		35.80	< 0.001
BMI (kg/m^2^): <18.5	67 (6.95)	161(43.05)	14.12	< 0.001	55(6.23)	173(38.02)		33.60	< 0.001
parity: primiparous	609 (63.17)	339(90.64)	18.01	< 0.001	550 (62.29)	398(87.47)		20.25	< 0.001
conception mode: assisted reproductive technology	52(5.39)	56 (14.97)	16.32	< 0.001	46(5.21)	62(13.63)		31.96	< 0.001
pregnancy complications	44(4.56)	63(16.84)	13.52	< 0.001	41(4.64)	66(14.51)		18.32	< 0.001
delivery mode: cesarean	391 (40.56)	331(88.50)	18.58	< 0.001	370(41.90)	352(77.36)		27.60	< 0.001
poor sleep quality	299 (31.02)	318 (85.03)	53.16	< 0.001	303 (34.31)	314 (69.01)		12.42	< 0.001
poor family support	337 (34.96)	302 (80.75)	36.32	< 0.001	334 (37.83)	305 (67.03)		10.31	< 0.001
fear of the adverse impact of chest CT examination	560 (58.09)	333 (89.03)	21.54	< 0.001	488 (55.27)	405 (89.01)		31.23	< 0.001
fear of adverse impact of disrupted scheduled pregnant inspection	453 (46.99)	247 (66.04)	6.40	0.013	410 (46.43)	290 (63.74)		5.70	0.018
worrying about difficulty in hospitalization	280 (29.05)	158 (42.25)	3.20	0.070	292 (33.07)	223 (49.01)		4.70	0.028
fear of being infected COVID-19	656(68.05)	360 (96.26)	27.18	< 0.001	597 (67.61)	419 (92.09)		26.56	< 0.001
fear of losing job after delivery	463 (48.03)	247 (66.04)	5.65	0.016	380 (43.04)	330 (72.53)		8.56	0.007
lack of knowledge of COVID-19	434 (45.02)	281 (75.13)	15.04	< 0.001	364 (41.22)	351 (77.14)		16.05	< 0.001
severe pain	435 (45.12)	228(60.96)	3.72	0.049	389(44.05)	274 (60.21)		7.52	0.046

### Univariate analysis of factors of postpartum depression

Compared with non-depressed puerperal women (EPDS < 13), postpartum depression was significantly higher in pregnant women who were younger than 35 years, fear risk of COVID-19 infection (%), shortage of knowledge on COVID-19, a lower education level, a middle annual household income, a parity of nullipara, assisted reproductive technology, disrupted the scheduled pregnant inspection, poor family support, poor sleep quality, pregnancy complications, and with a BMI < 18.5 kg/m^2^. The detailed univariate analysis of factors of postpartum depression was shown in Table 4.

**Table 4:** Univariate analysis of factors of postpartum depression [cases (%)]

item	Postpartum depression			
	EPDS < 13	EPDS ≥ 13	χ2	P
Total	1003 (74.96)	335 (25.04)	-	-
Age:<35	825(82.25)	323 (96.42)	20.32	< 0.001
Education: high school or below	58(5.78)	130 (38.81)	37.06	< 0.001
Income: low	130 (12.96)	279 (83.28)	23.36	< 0.001
BMI (kg/m^2^): <18.5	31(3.09)	97 (28.96)	30.59	< 0.001
Parity: primiparous	633 (63.11)	315 (94.03)	17.52	< 0.001
Conception mode: assisted reproductive technology	28 (2.79)	80 (23.88)	35.13	< 0.001
Pregnancy complications	29 (2.89)	78 (23.28)	30.16	< 0.001
Delivery mode: cesarean	429 (42.77)	293(87.46)	32.04	< 0.001
fear of being infected COVID-19	673 (67.10)	309 (92.24)	27.60	< 0.001
fear of the adverse impact of chest CT	352 (35.09)	255 (76.12)	29.84	< 0.001
poor sleep quality	291 (29.01)	245 (73.13)	33.85	< 0.001
poor family support	301 (30.01)	242 (72.24)	30.46	< 0.001
lack of knowledge of COVID-19	281 (28.02)	252 (75.22)	35.31	< 0.001
severe pain	352 (35.09)	168 (50.15)	4.72	0.029
worrying about difficulty in hospitalization	282 (28.12)	141 (42.09)	3.20	0.074
fear of losing job position after delivery	243 (24.23)	212 (36.01)	3.28	0.076
fear of adverse impact of disrupted scheduled pregnant inspection	251(25.02)	117(34.93)	3.43	0.077

### Multivariate linear regression analysis of factors of postpartum depression

Multivariate linear regression was performed to explore the factors most associated with postpartum depression. The significant variables of univariate analysis were taken as independent variables and EPDS score as dependent variables. Of the probable factors related to the development of postpartum depression that were entered into multivariate logistic regression analysis, five factors were found to be significant. The general demographic background characteristics were determined to be significant to the development of postpartum depression, which included younger than 35 years, a lower education level, a middle annual household income and a BMI < 18.5 kg/m^2^. In addition, the pregnancy characteristics were also significant to the development of postpartum depression. The postpartum depression risk was higher among women who were with a parity of nullipara, assisted reproductive technology, disrupted the scheduled pregnant inspection, and pregnancy complications. Finally, the attitudes towards COVID-19 were associated with anxiety status. Those with relatively more knowledge about COVID-19, a rational risk perception and enough family care were less likely to be postpartum depression.

A subgroup analysis was conducted to explore risk factors specifically associated with depressive symptoms during the long-term normal pandemic prevention of COVID-19. The occurrence of depressive symptoms was taken as the dependent variable, and logistic regression analysis was performed. The results showed that the significant risk factors for postpartum depression symptoms during the long-term normal pandemic prevention of COVID-19 included fear of being infected ([odds ratio] OR = 3.924, 95% [confidence interval] CI: 1.692–8.586, P = 0. 001), low education level (OR = 2.608, 95%CI:1.231–6.549, P = 0.034), pregnancy complications (OR = 2.524,95%CI: 1.086–6.242, P = 0.042), poor family care (OR = 2.456, 95%CI: 1.068–5.698, P = 0.032), and few knowledge of COVID-19 (OR = 2.458, 95%CI:1.012–6.032, P = 0.036). Multifactor regression analysis of influencing factors of postpartum depression was shown in Table 5.

**Table 5:** Multifactor regression analysis of influencing factors of postpartum depression

factors	β	SE	Wald χ2	P	OR	95% CI
age below 35	0.904	0.456	3.945	0.049	2.469	1.008–6.015
low education level	1.308	0.418	6.021	0.034	2.608	1.231–6.549
low annual household income	0.896	0.428	4.328	0.039	2.446	1.055–5.683
BMI < 18.5 kg/m^2^	1.106	0.437	4.980	0.041	2.538	1.120–6.280
primiparous	1.138	0.423	5.175	0.037	2.527	1.143–6.116
assisted reproductive technology	0.901	0.442	4.135	0.044	2.457	1.032–5.849
pregnancy complications	1.226	0.426	5.218	0.042	2.524	1.086–6.242
cesarean	1.064	0.434	4.676	0.043	2.491	1.058–6.045
poor sleep quality	1.148	0.425	7.390	0.016	3.190	1.380–7.140
poor family support	0.956	0.436	4.423	0.032	2.456	1.068–5.698
fear of being infected	1.340	0.415	10.356	0.001	3.924	1.692–8.586
lack of knowledge of COVID-19	0.901	0.452	3.908	0.036	2.458	1.012–6.032
fear of the adverse impact of chest CT	0.448	0.435	0.959	0.886	1.380	0.556–3.326
severe pain	0.527	0.468	1.782	0.181	1.868	0.742–4.687

## Discussion

As a special population, pregnant women were more susceptible to psychological problems such as anxiety and depression due to changes in endocrine hormones, weakened psychological responses and the continuation of negative emotions. The global incidence of postpartum depression was estimated to be approximately 10–20%, depending on the different sample and methodology. The incidence of postpartum depression in China was 12.2% [31, 32]. Pregnant women during the long-term normal prevention of COVID-19 pandemic period were more likely to suffer from anxiety and depression due to receiving more information about the pandemic and difficulties in seeking medical care. The depressive symptom among the pregnant women was about 26–56.3% during COVID-19 pandemic, including 40% in US [33], 37% in Canada [34], 56.3% in Türkiye [35] and 29.6% in China [21]. In this study, the results showed that the incidence of prenatal anxiety (SAS score ≥ 50) was 27.95% (374 cases), that of prenatal depression (SDS score ≥ 0.5) was 34.01% (455 cases), and postpartum depression (EPDS score ≥ 0.5) was 25.04% (335 cases). The anxiety and depression incidences in this study were higher than those reported in Shenzhen [36]. Both the prenatal SAS score (r = 0.635, P < 0.001) and prenatal SDS score (r = 0.738, P < 0.001) were related to postpartum depression, which were a litter lower than that during COVID-19 outbreak, but still higher than that before COVID-19 outbreak.

All those strongly indicated that incidence of anxiety and depression in perinatal mothers had significantly increased during the long-term normal prevention of COVID-19 pandemic period. Only few research showed that the pregnant women had an advantage of facing mental problems caused by COVID-19 during the COVID-19 pandemic, showing fewer anxiety and depression [37]. The attitudes towards COVID-19 were associated with anxiety status. Those with relatively more knowledge about COVID-19 and with a rational risk perception, were less likely to be anxious [38]. Additionally, positive attitudes towards online medical consultation demonstrated a protective feature from anxiety, whereas those who opted for psychological consultation showed the opposite effect.

The main risk factor included young age, low education level, low annual household income, BMI < 18.5 kg/m^2^, primiparous, assisted reproductive technology, pregnancy complications, cesarean, poor sleep quality, poor family support, fear of being infected and few knowledge of COVID-19. Pregnant women who were younger than 35 years and primiparous seemed to be more vulnerable to develop depressive symptoms because most of them had no experience of giving birth and they were more likely get nervous. Those pregnant women with assisted reproductive technology were easily to get anxiety and depression because the pregnancy process were more difficult for them and they were more worried about their babies’ health. We found that pregnant women with low annual household income and fear losing job positions were likely to report anxiety because family income pressure would make them nervous. The annual family income factor was different with previous research which reported that pregnant women from middle-level income families were about half as likely to report anxiety than were those earning an extremely high or low wage [39]. The attitudes towards COVID-19 were associated with anxiety and depression status. Those with relatively more knowledge about COVID-19 and with a rational risk perception were less likely to be anxious because they were not too nervous about epidemic control. There was a significant relationship between pregnant women’s concerns on the adverse effect of COVID-19 symptoms of depression. The pregnant women who were fear of adverse impact of disrupted scheduled pregnant inspection or worrying difficulty in hospitalization would develop severity of depression. Family support was also an important factor to reduce the anxiety and depression of pregnant women.

How to effectively prevent the occurrence of maternal anxiety and depression was one of the important issues for special groups of people during the long-term normal prevention of COVID-19 pandemic [40]. According to the factors influencing maternal mental health in different periods before and after delivery, appropriate psychological intervention was indispensable. It was suggested to provide pregnant women with necessary nutritional support, arrange sleep time reasonably, and avoid circadian rhythm disorders. It was also necessary to reduce exposure to COVID-19 information and reduce the negative emotions and actively respond to the psychological crisis caused by COVID-19. Online psychological counseling hotline could be widely carried out to avoid unnecessary physical and mental trauma and malignant adverse events [41, 42].

The COVID-19 situation is rapidly changing. Now, the coronavirus pandemic outbreak has almost been ended all over the world. But there are some sporadic cases who are re-infected with COVID-19 in China, only that the symptom is less severe than that during the pandemic outbreak. The findings in this study are still valid for pregnant women preventing from developing anxiety and depression. Psychological intervention and corresponding public health measures are necessary and requires multidisciplinary cooperation. These interventions include encouraging the pregnant women to get the information from authoritative sources, understanding correctly the susceptibility to disease, perceiving the risk rationally and using online counselling. Besides, preventive measures, long-term normal mental health screening, and long-term normal medical checkups are needed with the goal to reduce the risk of depression in this vulnerable population [43, 44].

One of the strengths of this study is that a multivariable logistic regression analysis method was used to exam the corresponding risk factors of anxiety and depression. In addition, the study includes a sufficient sample size to provide sufficient statistical power. However, there are several limitations in this study. First, as a majority of the women are primiparous, that itself is a cause for anxiety especially during perinatal period. Another limitation is that the participants are from only one hospital, resulting that the findings may not be representative of the entire population of pregnant women in China. Finally, as 53.96% women have undergone caesarean section (elective or emergency), pregnant women with caesarean section history are probably easy to develop anxiety or depression.

## Conclusions

In conclusion, the incidences of postpartum depression among perinatal women during the long-term normal prevention of COVID-19 pandemic period were a little lower than those during the COVID-19 outbreak period, but still twice as high as those before the COVID-19. The postpartum depression was associated with the degree of prenatal anxiety and depression. Pregnant women who were younger than 35 years, in middle household income, lower education level, underweight before pregnancy, primiparous, and fear of being infected were at increased risk for developing anxiety and depression during the long-term normal prevention of COVID-19 pandemic. Intervention should be taken to reduce the depression of pregnant women.

## Data Availability

The authors confirm that all the data supporting the findings of this study are included in this published article.

## References

[1] Wang C, Horby PW, Hayden FG, Gao GF (2020). A novel coronavirus outbreak of global health concern. The lancet.

[2] Huang C, Wang Y, Li X, Ren L, Zhao J, Hu Y, Zhang L, Fan G, Xu J, Gu X (2020). Clinical features of patients infected with 2019 novel coronavirus in Wuhan, China. The lancet.

[3] Organization WH. Clinical management of severe acute respiratory infection (SARI) when COVID-19 disease is suspected: interim guidance, 13 March 2020. In.: World Health Organization; 2020.

[4] Hamzehgardeshi Z, Omidvar S, Amoli AA, Firouzbakht M (2021). Pregnancy-related anxiety and its associated factors during COVID-19 pandemic in iranian pregnant women: a web-based cross-sectional study. BMC Pregnancy Childbirth.

[5] Chmielewska B, Barratt I, Townsend R, Kalafat E, van der Meulen J, Gurol-Urganci I, O’Brien P, Morris E, Draycott T, Thangaratinam S (2021). Effects of the COVID-19 pandemic on maternal and perinatal outcomes: a systematic review and meta-analysis. The Lancet Global Health.

[6] Heinen A, Varghese S, Krayem A, Molodynski A. Understanding health anxiety in the COVID-19 pandemic. Int J Soc Psychiatry 2021:00207640211057794. 10.1177/00207640211057794 34823387

[7] Lebel C, MacKinnon A, Bagshawe M, Tomfohr-Madsen L, Giesbrecht G (2020). Elevated depression and anxiety symptoms among pregnant individuals during the COVID-19 pandemic. J Affect Disord.

[8] Firouzbakht M, Rahmani N, Sharif Nia H, Omidvar S (2022). Coping strategies and depression during the COVID-19 pandemic in pregnant women: a cross sectional study. BMC Psychiatry.

[9] Yan W, Wang X, Kuang H, Chen Y, Baktash MB, Eskenazi B, Ye L, Fang K, Xia Y (2020). Physical activity and blood pressure during pregnancy: mediation by anxiety symptoms. J Affect Disord.

[10] Kotabagi P, Fortune L, Essien S, Nauta M, Yoong W (2020). Anxiety and depression levels among pregnant women with COVID-19. Acta Obstet Gynecol Scand.

[11] Tomfohr-Madsen LM, Racine N, Giesbrecht GF, Lebel C, Madigan S (2021). Depression and anxiety in pregnancy during COVID-19: a rapid review and meta-analysis. Psychiatry Res.

[12] Van den Bergh BR, Dahnke R, Mennes M (2018). Prenatal stress and the developing brain: risks for neurodevelopmental disorders. Dev Psychopathol.

[13] Effati-Daryani F, Zarei S, Mohammadi A, Hemmati E, Ghasemi Yngyknd S, Mirghafourvand M (2020). Depression, stress, anxiety and their predictors in iranian pregnant women during the outbreak of COVID-19. BMC Psychol.

[14] Nayak D, Karuppusamy D, Maurya DK, Kar SS, Bharadwaj B, Keepanasseril A (2021). Postpartum depression and its risk factors in women with a potentially life-threatening complication. Int J Gynecol Obstet.

[15] Aly HM, Nemr NA, Kishk RM, bakr Elsaid NMA (2021). Stress, anxiety and depression among healthcare workers facing COVID-19 pandemic in Egypt: a cross-sectional online-based study. BMJ open.

[16] Yue C, Liu C, Wang J, Zhang M, Wu H, Li C, Yang X (2021). Association between social support and anxiety among pregnant women in the third trimester during the coronavirus disease 2019 (COVID-19) epidemic in Qingdao, China: the mediating effect of risk perception. Int J Soc Psychiatry.

[17] Hamzehgardeshi Z, Omidvar S, Amoli AA, Firouzbakht M. Pregnancy-related anxiety and its associated factors during COVID-19 pandemic in iranian pregnant women: a web-based cross-sectional study. BMC Pregnancy Childbirth. 2021;21(1):208. 10.1186/s12884-021-03694-9 PMC7957463 33722198

[18] Gildner TE, Laugier EJ, Thayer ZM (2020). Exercise routine change is associated with prenatal depression scores during the COVID-19 pandemic among pregnant women across the United States. PLoS ONE.

[19] Sut HK, Kucukkaya B. Anxiety, depression, and related factors in pregnant women during the COVID-19 pandemic in Turkey: A web‐based cross‐sectional study. Perspect Psychiatr Care. 2021;57(2):860–8. 10.1111/ppc.12627 PMC7537279 32989798

[20] Ravaldi C, Ricca V, Wilson A, Homer C, Vannacci A (2020). Previous psychopathology predicted severe COVID-19 concern, anxiety, and PTSD symptoms in pregnant women during “lockdown” in Italy. Arch Women Ment Health.

[21] Wu Y, Zhang C, Liu H, Duan C, Li C, Fan J, Li H, Chen L, Xu H, Li X (2020). Perinatal depressive and anxiety symptoms of pregnant women during the coronavirus disease 2019 outbreak in China. Am J Obstet Gynecol.

[22] Wu Y, Zhang C, Liu H, Duan C, Huang H. Perinatal depressive and anxiety symptoms of pregnant women along with COVID-19 outbreak in China. Am J Obstet Gynecol 2020. 10.1016/j.ajog.2020.05.009 PMC7211756 32437665

[23] King LS, Feddoes DE, Kirshenbaum JS, Humphreys KL, Gotlib IH. Pregnancy during the pandemic: the impact of COVID-19-related stress on risk for prenatal depression. Psychol Med 2021:1–32. 10.1017/S003329172100132X PMC8047399 33781354

[24] Kuang G, Meng X, Wang Y, Xu R, Zhang M. Development and psychometric evaluation of self-management scale for pregnant woman with gestational diabetes mellitus in China. Nurs Open. 2022;9(3):1757–65. 10.1002/nop2.1202 PMC8994948 35224873

[25] Ghazanfarpour M, Bahrami F, Fakari FR, Ashrafinia F, Abdi F. Prevalence of anxiety and depression among pregnant women during the COVID-19 pandemic: a meta-analysis. J Psychosom Obstet Gynecol 2021(2):1–12. 10.1080/0167482X.2021.1929162 34165032

[26] Duranku F, Aksu E. Effects of the COVID-19 pandemic on anxiety and depressive symptoms in pregnant women: a preliminary study. J Maternal-Fetal Neonatal Med 2020(10223):1–7. 10.1080/14767058.2020.1763946 32419558

[27] Zhang Y, Muyiduli X, Wang S, Jiang W, Wu J, Li M, Mo M, Jiang S, Wang Z, Shao B (2018). Prevalence and relevant factors of anxiety and depression among pregnant women in a cohort study from south-east China. J reproductive infant Psychol.

[28] January J, Chimbari MJ (2018). Study protocol on criterion validation of Edinburgh postnatal depression scale (EPDS), Patient Health Questionnaire (PHQ-9) and centre for Epidemiological Studies-Depression (CES-D) screening tools among rural postnatal women; a cross-sectional study. BMJ open.

[29] Zhang M, Chen W, Liu C, Sui J, Wang D, Wang Y, Meng X, Wang Y, Yue C (2021). Nursing-sensitive quality indicators for pernicious placenta previa in obstetrics: a Delphi study based across chinese institutions. Nurs Open.

[30] Liu JH, Hung PY, Alberg AJ, Hair NL, Whitaker KM, Simon J, Taylor SK (2021). Mental health among pregnant women with COVID-19-related stressors and worries in the United States. Birth-Issues in Perinatal Care.

[31] Fu CW, Liu JT, Tu WJ, Yang JQ, Cao Y (2015). Association between serum 25-hydroxyvitamin D levels measured 24 hours after delivery and postpartum depression. Bjog-an Int J Obstet Gynecol..

[32] Patabendige M, Gamage MM, Weerasinghe M, Jayawardane A (2020). Psychological impact of the COVID-19 pandemic among pregnant women in Sri Lanka. Int J Gynecol Obstet.

[33] King LS, Feddoes DE, Kirshenbaum JS, Humphreys KL, Gotlib IH. Pregnancy during the pandemic: the impact of COVID-19-related stress on risk for prenatal depression. Psychol Med 2021:1–11. 10.1017/S003329172100132X PMC8047399 33781354

[34] Clab C, Mkb D, Mbab C, Tmbd E, Ggbd E (2020). Elevated depression and anxiety symptoms among pregnant individuals during the COVID-19 pandemic. J Affect Disord.

[35] Sut HK, Kucukkaya B (2020). Anxiety, depression, and related factors in pregnant women during the COVID-19 pandemic in Turkey: a web‐based cross‐sectional study. Perspect Psychiatr Care.

[36] Wu F, Lin W, Liu PY, Zhang MY, Huang SB, Chen CY, Li QS, Huang WK, Zhong CY, Wang YY (2021). Prevalence and contributory factors of anxiety and depression among pregnant women in the post-pandemic era of COVID-19 in Shenzhen, China. J Affect Disord.

[37] Zhou YJ, Shi H, Liu ZK, Peng SX, Wang RX, Qi L, Li ZZ, Yang JZ, Ren YL, Song XL et al. The prevalence of psychiatric symptoms of pregnant and non-pregnant women during the COVID-19 epidemic. Translational Psychiatry. 2020;10(1):319 10.1038/s41398-020-01006-x PMC7501755 32950999

[38] Lee T-Y, Zhong Y, Zhou J, He X, Kong R, Ji J (2021). The outbreak of coronavirus disease in China: risk perceptions, knowledge, and information sources among prenatal and postnatal women. Women Birth.

[39] Liu X, Chen M, Wang Y, Sun L, Qi H. Prenatal anxiety and obstetric decisions among pregnant women in Wuhan and Chongqing during the COVID outbreak: a cross ectional study. BJOG An International Journal of Obstetrics & Gynaecology. 2020;127:1229–440. 10.1111/1471-0528.16381 PMC7362035 32583536

[40] Zheng Z, Zhang RX, Liu T, Cheng P, Zhou YH, Lu WC, Xu GY, So KF, Lin KG. The psychological impact of the Coronavirus Disease 2019 pandemic on pregnant women in China. Front Psychiatry. 2021;12:628835. 10.3389/fpsyt.2021.628835 PMC8282994 34276429

[41] Lin W, Wu B, Chen B, Lai GY, Huang SB, Li SL, Liu KF, Zhong CY, Huang WK, Yuan SX (2021). Sleep conditions associate with anxiety and depression symptoms among pregnant women during the epidemic of COVID-19 in Shenzhen. J Affect Disord.

[42] Firouzbakht M, Rahmani N, Nia HS, Omidvar S. Coping strategies and depression during the COVID-19 pandemic in pregnant women: a cross sectional study. BMC Psychiatry. 2022;22(1):153. 10.1186/s12888-022-03792-8 PMC8886336 35232424

[43] Liu X, Chen M, Wang Y, Sun L, Zhang J, Shi Y, Wang J, Zhang H, Sun G, Baker PN (2020). Prenatal anxiety and obstetric decisions among pregnant women in Wuhan and Chongqing during the COVID-19 outbreak: a cross-sectional study. Bjog-an Int J Obstet Gynecol.

[44] Bo HX, Yang Y, Chen J, Zhang M, Li YL, Zhang DY, Li Y, Li R, Cheung T, Ng CH (2021). Prevalence of depressive symptoms among pregnant and Postpartum Women in China during the COVID-19 pandemic. Psychosom Med.

